# Current Status and Prospects of Spontaneous Peritonitis in Patients with Cirrhosis

**DOI:** 10.1155/2020/3743962

**Published:** 2020-07-06

**Authors:** Yong-Tao Li, Jian-Rong Huang, Mei-Lian Peng

**Affiliations:** ^1^State Key Laboratory for Diagnosis and Treatment of Infectious Diseases, The First Affiliated Hospital, College of Medicine, Zhejiang University, Hangzhou, 310003 Zhejiang Province, China; ^2^Zhejiang Provincial People's Hospital, Hangzhou, 310014 Zhejiang Province, China

## Abstract

Spontaneous bacterial peritonitis (SBP) is a common cirrhotic ascites complication which exacerbates the patient's condition. SBP is caused by gram-negative bacilli and, to a lesser extent, gram-positive cocci. Hospital-acquired infections show higher levels of drug-resistant bacteria. Geographical location influences pathogenic bacteria distribution; therefore, different hospitals in the same country record different bacteria strains. Intestinal changes and a weak immune system in patients with liver cirrhosis lead to bacterial translocation thus causing SBP. Early diagnosis and timely treatment are important in SBP management. When the treatment effect is not effective, other rare pathogens should be explored.

## 1. Introduction

Spontaneous bacterial peritonitis (SBP) is a common complication in patients with liver cirrhosis and is recorded in 10–30% of hospitalized patients with cirrhotic ascites leading to sepsis or even death [1–4]. Studies show that bacterial translocation plays a key role in the occurrence and development of SBP [5, 6]. Bacterial translocation is caused by disorder of gut microflora, increased intestinal permeability, and host immunodeficiency [7, 8]. Although gram-negative bacilli are the main cause of SBP, infections due to gram-positive bacteria drug-resistant bacteria have been reported [9–11]. Therefore, it is important to understand the epidemiology and pathogenesis of SBP and develop effective therapy approaches.

## 2. Epidemiology

Geographical location affects SBP pathogen distribution with variations recorded among different hospitals in the same country. Gram-negative bacilli are the main SBP-causing pathogens, but infections of gram-positive cocci [12, 13], fungi, and some other rare pathogens cannot be ignored [14–18]. Increased use of broad-spectrum antibiotics and prophylactic quinolones has led to the emergence of multidrug-resistant bacteria, especially in hospital-acquired infections [19–22]. Only 50-60% of SBP patients have positive ascites culture; therefore, pathogen identification is challenging [23]. These limitations hamper development of effective anti-infection therapy.

### 2.1. Asia

Li et al. [24] retrospectively analyzed 288 Chinese patients with spontaneous peritonitis from 2011 to 2013 and isolated 306 pathogenic bacteria, among which gram-negative bacteria, gram-positive bacteria, and fungi accounted for 58.2%, 27.8%, and 2.9% of the isolates. The main pathogenic bacteria were *Escherichia coli*, *Klebsiella pneumoniae*, *Enterococcus,* and *Staphylococcus aureus*. Of the 306 pathogenic bacteria, 99 cause nosocomial infections and 207 were community-acquired and play a role in other infection pathogenesis. *Escherichia coli* and *K. pneumoniae* produce more broad-spectrum *β*-lactamase in nosocomial infections compared with nonnosocomial infections. Piperacillin/tazobactam combination is a more effective therapy for nonhospital infections than nosocomial infections caused by *E. coli*. The authors reported that the pathogenic bacteria causing abdominal infection in patients with liver cirrhosis were mainly gram-negative, and the drug resistance rate of nosocomial infection was significantly higher compared with the rate for nonnosocomial infection.

In another retrospective study, Ding et al. [25] analyzed the etiology of 334 Chinese patients with SBP from 2012 to 2016 and arrived at a similar conclusion. A total of 334 pathogenic bacteria were isolated, including 178 gram-negative bacteria and 138 gram-positive bacteria. The main pathogens were *E. coli*, *K. pneumoniae*, and *Enterococcus faecium*. The proportion of *Enterococci* in patients with hospital-acquired SBP was significantly higher than in those with community-acquired SBP. Pathogens isolated from nosocomial infections showed significantly higher resistance to first-line recommended drugs and were associated with poor prognosis.

In a retrospective cohort study in South Korea, Cheong et al. [21] analyzed the microbial characteristics of 236 patients with SBP from 2000 to 2007: *E. coli* accounted for 43.2%, *Klebsiella* accounted for 14.0% while *Streptococcus* accounted for 9.8% of the total bacteria population. The resistance rate of G^−^ to third generation cephalosporins and quinolones for hospital-acquired infections was significantly higher compared with that for community-acquired infections. In another study, Choi et al. [15] found 43 cases of SBP caused by *Aeromonas aerobicus* as a result of weather changes between 1997 and 2006. Hwang et al. [26] reported that *Candida* infection was the main causative agent of fungal spontaneous peritonitis in Korea from 2000 to 2005.

### 2.2. Europe

In a Spanish retrospective study from 2001 to 2009, 34.6% of the 200 SBP patients showed community-acquired infections while 26.8% of these infections were hospital acquired. The third-generation cephalosporin resistance rate was 7.1% for the community-acquired infections and 40.9% for the hospital-acquired infections. These drug-resistant cases were mainly a result of gram-negative bacilli and *Enterococci* that produce extended-spectrum *β*-lactamases. Previous use of cephalosporins, diabetes, upper gastrointestinal bleeding, and nosocomial-acquired infections are risk factors for the development of drug-resistant bacterial infections [27]. Fernandez et al. [28] analyzed bacterial infection in 507 Spanish patients with liver cirrhosis and ascites admitted to hospital during 2005–2007 and 2010–2011 in a prospective study. 35% of hospital-acquired patients had higher number of drug-resistant strains compared with those with community-acquired infections (4%). Moreover, SBP mortality caused by drug-resistant bacteria was significantly higher.

Friedrich et al. [29] retrospectively analyzed the etiology of the first occurrence of SBP in 311 German patients with liver cirrhosis from 2007 to 2013. Gram-positive bacteria accounted for 47.8% of the total infections, gram-negative bacteria accounted for 44.9% while fungi accounted for 7.2% of the infections. In this study, *Enterobacter*, *Enterococcus*, and *Staphylococcus* were the most common isolates. Third-generation cephalosporins were effective in 70.2% of non-hospital-acquired SBP patients and in 56.3% of hospital-acquired SBP patients. In another prospective study from Germany, Lutz et al. [30] analyzed 86 German SBP patients from 2012 to 2016 and obtained similar results. *E. coli*, *Klebsiella*, *Enterococcus*, and *Streptococcus* were the most common isolates. The resistance rate of nosocomial bacteria was higher than that of healthcare-related bacteria.

Bert et al. [31] analyzed 95 cases of hospital-acquired and community-acquired bacterial peritonitis in France from 1998 to 1999. A total of 78 pathogenic bacteria were isolated, of which 34 were *Streptococcus* spp. and 23 were *E. coli*. Streptococci are more common in community-acquired infections while gram-negative bacteria are more common in hospital-acquired infections. Another prospective observational study in France in 2005, involving 331 patients with SBP at 25 medical centers, revealed 222 gram-negative bacilli, mainly *E. coli*, *Enterobacter*, *K. pneumoniae*, and *P. aeruginosa*; 148 gram-positive cocci, mainly *Streptococcus*, *Enterococcus faecalis*, *Enterococcus faecium*, and *Staphylococcus aureus* while all 19 strains of fungi were *Candida albicans* [32]. Imipenem is an effective treatment for *P. aeruginosa* hospital-acquired infections [32].

Piroth et al. [33] retrospectively analyzed 114 strains of SBP in five hospitals in France from 2006 to 2007. *Staphylococci* and *E. coli* were the most common pathogens. Notably, 28% patients infected by the *E. coli* strain showed resistance to amoxicillin+clavulanic acid, and 27% of patients infected with *S. aureus* were resistant to methicillin. An observational study carried out in France in 2010 and 2011 showed that of the 57 confirmed SBP cases, gram-positive cocci (64.9%) were the main causative pathogens, including coagulase-negative *Staphylococci*, *Enterococci*, *Streptococci*, *Staphylococcus aureus*, and *Streptococcus pneumoniae* [13]. Another study on SBP patients in France reported that gram-positive bacteria were the dominant strains, accounting for 70% of nosocomial infections [34].

Gunjaca and Francetić [35] prospectively studied 108 cases of cirrhosis in Croatia, where SBP prevalence was 21% and the mortality was 26%. The pathogens causing SBP were mainly gram-negative bacteria such as *E. coli*, methicillin-resistant *S. aureus* (MRSA), and *Acinetobacter*.

Alexopoulou et al. [36] retrospectively carried out a study on 47 SBP patients in Greece from 2008 to 2011. Twenty-eight patients had medically related infections and 15 were treated with quinolone prophylaxis. Gram-positive coccus was the most commonly isolated pathogen. Nine isolates were multidrug-resistant bacteria, including *K. pneumoniae*-producing carbapenemase and *E. coli-* and *P. aeruginosa*-producing ultrabroad spectrum *β*-lactamase. Higher number of gram-negative bacteria was reported in hospital-associated infections compared with gram-positive cocci. Another Greek prospective study from 2012 to 2014 included 130 SBP patients with a 30-day follow-up. The results showed that gram-positive cocci (GPC) were the causative agents for half of the cases. Multidrug-resistant (MDR) strains comprised 20.8% of the total cases while 10% were extensively drug resistant (XDR). Drug-resistant bacteria showed a significant increase in mortality rates [37].

### 2.3. America

Chaulk et al. [38] retrospectively analyzed 192 Canadian SBP patients from 2003 to 2011. Among them, 77 patients had culture-positive infection with gram-positive bacteria causing 57% of these cases. The antibiotic resistance rate was 8% in community-acquired infections and 41% in hospital-acquired infections (Table 1).

Ardolino et al. [39] retrospectively studied 160 SBP cases in the United States from 2005 to 2015. This study reports that gram-negative bacteria were mainly *E. coli*. The sensitivity rate to ceftriaxone was 71%. Gram-positive cocci including *Enterococci*, *Streptococcus*, and *Staphylococcus* accounted for 37.5% of the cases. 71% of *Enterococci* were resistant to vancomycin, and MRSA accounted for 80% of the infections.

Reddy et al. [40] reported a rare case of SBP caused by the *Salmonella enteritis* group b in a patient with liver cirrhosis in the United States. Wu and Giri [41] first reported a case of SBP caused by *Haemophilus paraphilus*. Later, the patient also developed tuberculous peritonitis, a combination that had not been reported before. Emily and Maraj [42] reported cases of SBP with *Lactobacillus* as the pathogen. *Lactobacillus paracasei* was isolated from the abdominal cavity of a 73-year-old American man with liver cirrhosis. This strain was resistant to carbapenem antibiotics. Further, the patient eventually developed hepatorenal syndrome and succumbed to acute renal failure. Toyoshima et al. [43] reported SBP cases caused by *Listeria monocytogenes* in two patients with liver cirrhosis in Brazil. Third-generation cephalosporins are not effective for *Listeria* infections.

### 2.4. Africa

Oladimeji et al. [44] conducted a retrospective analysis of 31 patients with ascites in Nigeria from 2009 to 2010. In these SBP patients, the main pathogens were *E. coli* and *Klebsiella*. The gram-positive bacteria implicated in SBP infections were mainly *Streptococcus* and *Staphylococcus aureus*. Zaki et al. [45] explored the bacterial and fungal causes of SBP in an Egyptian population comprising 100 SBP patients. In this population, the pathogens were mainly gram-positive coccus (48.8%), gram-negative bacillus (12.2%), and 7.3% were *Mycobacterium tuberculosis*. Mohamed et al. [46] performed SBP screening on 3000 cirrhosis patients with ascites and pleural effusion in Egypt. SBP prevalence in patients with cirrhosis was reported to be 1.6% with the main causative pathogens being *E. coli* and *K. pneumoniae.*

## 3. Pathogenesis

**Figure 1:**
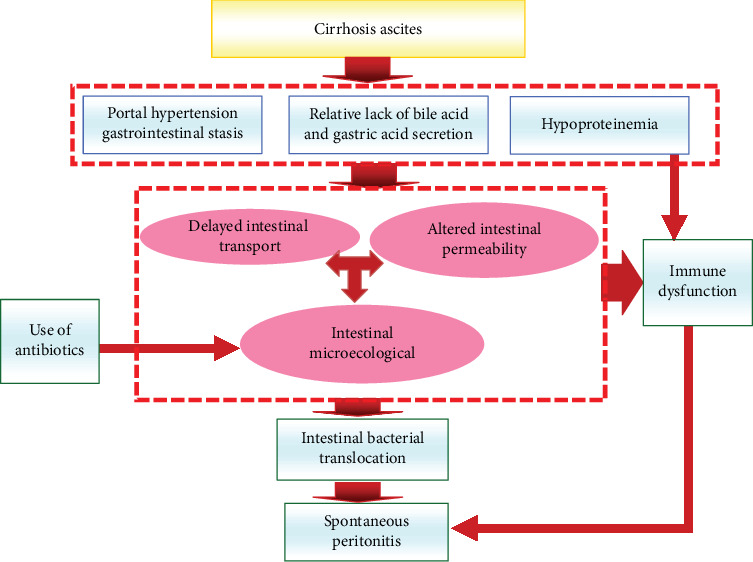
The pathogenesis of spontaneous peritonitis.

Intestinal flora is considered as an important component of the intestinal barrier [47]. Changes to the gut microbiota are implicated in the SBP occurrence and progression [48–51]. Therefore, exploring the role of intestinal flora on SBP pathogenesis is the key in development of effective prevention and treatment strategies. For patients with liver cirrhosis, bacterial translocation (BT) as a result of intestinal gram-negative *Enterobacteriaceae* infections is the main cause of SBP occurrence and development [6, 52, 53]. Previous studies have shown that gastrointestinal stasis due to portal hypertension in patients with liver cirrhosis, intestinal bacterial overgrowth due to low levels of bile acid and gastric acid, delayed intestinal transport, altered intestinal permeability, and immune dysfunction promote BT and ultimately SBP [5, 7, 8] (Figure 1).

### 3.1. Small Intestinal Bacterial Overgrowth (SIBO)

Cirrhosis results in small intestinal bacterial overgrowth [54–56], especially in patients with ascites and SBP history [57]. Overgrowth of small intestinal bacteria is implicated in bacterial translocation and SBP [58]. In a previous study, Bauer et al. reported that small intestinal bacterial overgrowth (SIBO) in patients with cirrhosis has no effect on spontaneous bacterial peritonitis [59]. However, in a subsequent study, he carried out quantitative culturing of jejunal secretion in 53 cirrhosis patients with a 1-year follow-up. In his findings, he reported that SIBO was present in 59% of the cirrhosis patients he examined and was associated with systemic endotoxemia [60]. Fukui et al. [61] also reported an increase in gram-negative bacteria represented by *E. coli* resulting in high levels of lipopolysaccharides (LPS) and endotoxemia in patients with liver disease. BT or microbial translocation is defined as the migration of surviving microorganisms or bacterial products (i.e., bacterial LPS, peptidoglycans, and lipopeptides) from the intestinal lumen to the mesenteric lymph nodes and other external intestinal sites [62–66]. In addition, studies have shown that small bowel transport is significantly longer in patients with SIBO [67]. Animal experiments by Pérez-Paramo et al. [68] reported that intestinal overgrowth and severe impairment of intestinal permeability in cirrhotic rats with ascites cause bacterial translocation and SIBO was associated with insufficient intestinal motility. In recent studies, gastrointestinal stasis due to portal hypertension, relative lack of bile and gastric acid secretion, intestinal dyskinesia, and long-term use of broad-spectrum antibiotics in patients with liver cirrhosis are implicated in increased intestinal aerobic bacteria and colonic bacterial migration to the jejunum and duodenum. These changes further cause SIBO and promote BT, which is implicated in SBP prognosis in patients with liver cirrhosis [7]. Notably, the most common pathogenic microorganisms were isolated from the intestinal flora of cirrhotic ascites in SBP patients [69]. Interestingly, quantitative metagenomics analysis showed that some of the bacteria in SIBO were oral strains. Qin et al. [70] proposed that oral symbiotic bacteria in liver cirrhosis patients invaded the intestine as a result of bile secretion changes in these patients. The changes in bile secretion created a more favorable environment for the survival of foreign bacteria in the intestinal tract. Pardo et al. [54] also reported that cisapride increases BT from the oral cavity to the cecum. The use of cisapride in cirrhotic rats showed reduction of SIBO and occurrence of BT.

### 3.2. Altered Intestinal Permeability

The human intestinal mucosa mechanical barrier is the first barrier against BT and consists of intestinal epithelial cells and cell-to-cell connections [71–73]. The intestinal barrier system of intestinal epithelial cells prevents the transportation of a large number of bacteria and bacterial products; therefore, few bacteria and bacterial products reach the liver [74]. Tight junctions between cells are the key in maintaining integrity of the intestinal barrier, and reduction in density of these tight junctions impairs the function of the intestinal barrier [75, 76]. Assimakopoulos et al. [77] reported that expression levels of proteins associated with tight junctions in intestinal epithelial cells were lower in cirrhosis patients compared with patients with decompensated cirrhosis. Animal experiments [78] show that the intestinal mucosa of rats with liver cirrhosis shows signs of atrophy, shortening, and villus rupture. Capsule endoscopy studies show abnormal changes in the mucosa of the small intestine in cirrhosis patients [79] while pathological examination shows shortening and atrophy of the small intestine [80, 81]. However, Du Plessis et al. [82] reported that electron microscopy showed complete epithelial barriers in patients with decompensated cirrhosis, implying that the epithelial barrier was functionally altered but structurally normal in cirrhosis. The contrasting findings may be due to differences in methodology and the relatively small number of studies/patients [83]. Assimakopoulos et al. [84] performed duodenal biopsies on healthy controls and patients with cirrhosis and decompensated cirrhosis. In this study, patients with decompensated and decompensated cirrhosis had decreased intestinal mucosa mitosis and increased cell apoptosis compared with the control group. Intestinal permeability changes with progression of cirrhosis and occurrence of SIBO, with increased intestinal permeability of bacteria and their products resulting in BT [83, 85, 86]. Several studies report that cirrhosis and ascites patients have significantly high intestinal permeability, while the intestinal permeability of patients with Child–Pugh C is significantly higher than the permeability of those with Child–Pugh with A and B cirrhosis [87, 88]. For patients with SBP history, intestinal permeability is higher and can lead to severe sepsis complications [89, 90].

### 3.3. Delayed Bowel Transit

Studies show that liver cirrhosis changes intestinal motility [91]. Delayed movements of the small intestine can lead to SIBO and eventually cause BT [92]. A radiological examination by Kalaitzakis et al. [93] showed that intestinal transit time was prolonged in 38% patients with liver cirrhosis. Chen et al. [94] used a noninvasive hydrogen breath test and found that the intestinal transit time of patients with decompensated cirrhosis was significantly longer compared with that of patients with decompensated cirrhosis. Further, the intestinal transit time was positively correlated with the severity of cirrhosis [95]. The small intestine transit delay and SIBO interact are associated and activate each other [71]. Perez-Paramo et al. [68] reported that nonselective beta blocker (NSBB (propranolol)) treatment in cirrhotic animals significantly reduces portal vein pressure and accelerates intestinal transport. The rate of bacterial overgrowth and metastasis in liver cirrhosis cases is low; therefore, intestinal bacteria overgrowth is positively correlated with insufficient intestinal motility. Propranolol accelerates intestinal transport and reduces bacterial overgrowth and transfer rates. However, Mandorfer et al. [96] found that although NSBB can reduce the risk of portal vein pressure and esophageal varix bleeding in patients with liver cirrhosis, it can increase the rate of hemodynamic disorders and liver-renal syndrome in patients with liver cirrhosis and SBP. Animal experiment results show that cisapride accelerates the transit time, improves the permeability of the small intestine, and reduces BT [97].

### 3.4. Impaired Local and Systemic Immune Function

Although the intestinal immune system is the last line of defense in microbial invasion, it the most important line of defense against intestinal microbial invasion. The interaction between intestinal flora and mucosal immune system is dynamic and complex [98]. Under normal physiological conditions, the microbiome can maintain a delicate balance with the mucosal immune system, which is extremely important for the host health [99]. Changes in the intestinal microenvironment causes excessive growth of opportunistic pathogenic bacteria and the reduction of symbiotic bacteria in critically ill patients. The changes aggravate mucosal immune dysfunction, promote the increase of intestinal BT, and eventually lead to intestinal infection [100–103].

Bacteria occur in the intestinal lymphoid tissue but do not harm the body, as they are usually effectively cleared by phagocytes [104]. Damage to the body's defense mechanisms also promotes subsequent infection of fluid in the peritoneal cavity [54]. Immune disorders in patients with cirrhosis are known as cirrhosis-associated immune dysfunction (CAID) [105]. Cirrhosis-related immune dysfunction and immunodeficiency are dynamic and result from liver inflammation driven primarily by monocytes/macrophages. The liver's mononuclear-phagocytic system function in patients with cirrhosis is impaired, leading to a decrease in the body's immune function and opsonin activity in the ascites [106]. This further reduces the level of bacteria removal leading to the body's inability to effectively remove pathogenic bacteria eventually causing bacterial translocation and ultimately results in SBP. Phagocytosis of hepatic macrophages in cirrhosis patients is lower compared with that in the healthy control group and is correlated with the severity of liver disease [107–110]. In addition, severe malnutrition in patients with cirrhosis also affects their immune system. Diet and nutrition are key factors in host-microbe interactions while starvation adversely affects intestinal mucosal integrity, epithelial cell proliferation, and mucin and antimicrobial peptide synthesis. Hodin et al. [111] observed autophagy of Paneth cells in starved mice due lack of enteral nutrition and decreased expression of antibacterial products. The poor nutrition weakened the protective effect on BT, thereby causing BT. Therefore, improving the nutritional status of patients with advanced cirrhosis improves the body's immune function and reduces the BT and SBP incidences. Albumin is specifically synthesized in the liver and is implicated in a myriad of functions such as the binding and transport of substances, the regulation of endothelial function, antioxidant and clearance properties, and the regulation of inflammatory responses. Serum albumin levels are low in liver cirrhosis patients due to synthetic defects, and structural and functional changes due to posttranscriptional modifications hinder their ability to perform physiological functions [112, 113].

## 4. Treatment

For patients with decompensated liver cirrhosis, spontaneous peritonitis can lead to further decompensation and multiple organ failure; therefore, SBP therapy is important for these patients. However, current methods are limited to antibiotic treatment, which leads to increases in drug-resistant bacteria and nonclassical pathogen infections [9–11]. Therefore, understanding the mechanism of SBP development, antibiotic treatment, new adjuvant treatment methods, and multiple treatment coordination are needed to minimize the occurrence of infection, reduce bacterial resistance, and improve survival.

### 4.1. Antibiotic Treatment

If the patient is clinically suspected of developing SBP, ascites culture should be performed immediately along with initiation of antibiotic treatment to reduce complications and improve survival [114, 115]. Third-generation broad-spectrum cephalosporin, cefixime, is the first-line treatment option for out-of-hospital SBP infection, with a recommended dose of 2 g/8 h (6 g/day) for 5 days [116, 117], which can be extended to 7 days [118]. Fluoroquinolones have good oral bioavailability and can be used as therapy for uncomplicated SBP [119]. Third-generation cephalosporin antibiotics and quinolones have been used to control SBP infection with high levels of clinical efficacy. However, long-term application increases the risk of bacterial resistance and double infection. Notably, *Enterobacteriaceae* family shows increased resistance to cephalosporins, particularly in nosocomial infections [120, 121]. Long-term preventive norfloxacin treatment reduces the risk of gram-negative infections but increases the risk of hospital-acquired *Staphylococcal* infections [122]. Therefore, considering that the distribution of SBP varies with geographic region and the proportion of drug-resistant pathogens is high, when selecting first-line empirical antibiotic treatment, the epidemic situation of drug-resistant bacteria should be based on the local situation [10]. Piperacillin/tazobactam is the first-line treatment for nosocomial SBP infection in areas with low resistance. Meropenem is recommended in hospitals with a high positive rate of ESBLs produced by *Enterobacteria* [30]. In areas with high prevalence of MRSA and vancomycin-sensitive *Enterococcus* (VSE), a combination of meropenem and vancomycin or teicoplanin is recommended, while linezolid is recommended in case of vancomycin-resistant *Enterococcus* (VRE) [19]. In areas with high resistance to third-generation cephalosporins, meropenem combined with daptomycin can be used to improve patient survival of the nosocomial SBP [123]. If the ascites culture is positive, non-broad-spectrum antibiotics should be selected according to the drug sensitivity results to reduce the emergence of drug-resistant bacteria [115]. When antibiotic therapy fails in patients with spontaneous peritonitis, the possibility of fungal or other rare pathogens should be considered [14, 26, 124].

### 4.2. Gut Microecological Intervention

Intestinal bacteria are the main source of infections in patients with decompensated cirrhosis; therefore, norfloxacin is often used to clear the intestines for preventive treatment. However, antibiotic prevention can lead to increase in drug-resistant bacteria [125, 126]. Therefore, prevention is limited to a small number of patients with a high risk of infection. Probiotics can competitively inhibit adhesion to epithelial cells through competitive nutrients, reduce intestinal pH, and secrete antibacterial compounds to inhibit the growth of harmful pathogenic microorganisms. On the contrary, probiotics improve the intestinal mucosal barrier function and regulate the liver's natural killing of T lymphocytes [127]. Studies have reported that probiotics can reduce BT and effectively prevent the occurrence of hepatic encephalopathy [128]. Rat models with cirrhosis show that probiotics reduce BT, proinflammatory response status, formation of ascites, and oxidative damage in the ileum [129]. In a previous study, *Bifidobacterium* was shown to reduce the expression of proinflammatory chemokine receptors in the lymphocytes of mice with liver cirrhosis. Thus, the intestinal permeability of mice treated with *Bifidobacterium* was reduced while the liver function and inflammatory response improved [65]. The use of probiotics in liver-damaged rats alters the host's intestinal environment and reduces the occurrence of BTs [6, 130]. In a randomized double-blind controlled experiment, Gupta et al. [66] reported that the hepatic vein pressure gradient in the probiotic group was significantly lower compared with the propranolol group and that the addition of probiotics increased the effectiveness of propranolol treatment. However, a randomized controlled trial by Pande et al. [131] showed that the addition of probiotics to norfloxacin had no significant effect on SBP prevention in cirrhosis and ascites patients. Although more studies should be carried out needed to support the application of probiotic therapy in the prevention or management of SBP, previous studies report that probiotic therapy is effective in managing gastrointestinal diseases.

### 4.3. Immunity Therapy

In addition to intestinal targeting methods, immunotherapy methods have been developed to reduce the susceptibility of patients with decompensated cirrhosis to infection. In addition to antibiotics, albumin is a key therapy for SBP patients as it restores the immune function and improves survival [132]. Studies have found that infusion of human albumin reduces immunosuppression and the risk of infection in patients with acute decompensated cirrhosis [9, 133]. Combination of antibiotics and albumin significantly reduces serum and ascites cytokines and LPS levels in patients with SBP [134]. Caraceni et al. [135] evaluated 440 patients with decompensated liver cirrhosis who received standard treatment or standard treatment plus albumin. The 18-month survival rate of the treatment group was significantly higher compared with that of the standard treatment group. Sort et al. [136] randomly divided 126 patients with SBP; the mortality rate of the antibiotic plus albumin group was lower compared with that of the antibiotic group. Although the role of albumin is beneficial, not all patients with SBP can be treated with albumin, and patients with bile < 68.4 *μ*mol/L and creatinine < 88.4 *μ*mol/L cannot receive albumin treatment [136, 137]. Most patients with advanced liver cirrhosis are malnourished, which can easily lead to BT and SBP [138]. Patients with liver cirrhosis should optimize nutrition, avoid raw foods and coarse superfoods, limit sodium intake, eat small meals, and include 1.2-1.5 g of protein daily [139]. Cytokine treatments can improve the function of existing immune cells, significantly increase peripheral white blood cell counts, and improve the prognosis of patients with decompensated cirrhosis [140, 141]; however, more experimental and clinical evidence is needed.

## 5. Conclusion

**Figure 2:**
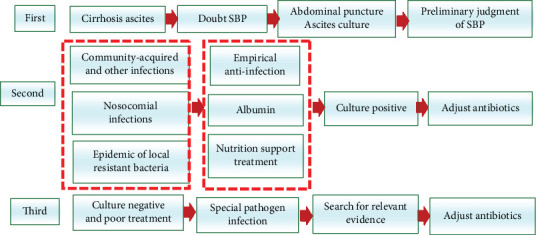
Treatment procedure of spontaneous peritonitis.

Spontaneous bacterial peritonitis causes high mortality rates and occurs in 7-31% of hospitalized patients with cirrhosis and ascites [142]. Patients susceptible to SBP need rigorous evaluation to optimize nutrition and avoid unnecessary drug treatment [12]. When patients with cirrhosis and ascites are hospitalized for gastrointestinal and parenteral diseases, ascites analysis should be performed whether symptoms are present or not. The long-term use of antibiotics has led to the emergence of multidrug-resistant bacteria and recent changes in the bacterial spectrum, including increased incidence of SBP associated with gram-positive cocci. Therefore, patients with cirrhosis and ascites should be monitored keenly and early diagnosis and treatment of SBP are important to prevent poor prognosis. A good understanding of the epidemiology of the region is the key to the correct choice of antibiotics. When encountering cases with poor treatment results, it is necessary to consider the possibility of other rare pathogens such as fungi and adjust the treatment strategy. Therapy approaches should include improved nutrition support to enhance the immunity of patients and comprehensive treatment should be considered for better results (Figure 2). SBP prevention should focus on stabilizing the intestinal environment, restoring the balance of intestinal flora, and reducing the occurrence of BT.

## Figures and Tables

**Table 1:** Pathogens associated with spontaneous peritonitis in cirrhosis.

Country/author/year	Pathogens	Type of study	G^−^	G^+^	HA SBP	CA SBP
China/Li et al./2011-2013	306	Retrospective	58.2%	27.8%	99	207
China/Ding et al./2102-2016	334	Retrospective	52.3%	41.3%	155	179
Korea/Cheong/2000-2007	236	Retrospective	72.9%	22.9%	126	110
Germany/Friedrich/2007-2013	114	Retrospective	44.9%	47.8%	—	—
France/Bert/1998-1999	78	Retrospective	44.9%	51.3%	39	39
France/Montravers/2005.1-2005.7	829	Prospective	41%	27%	540	289
France/Piroch/2010-2011	268	Prospective	34%	64.9%	109	159
Canada/Chaulk/2003-2011	77	Retrospective	27%	57%	52	25

G^−^: gram-negative bacteria; G^+^: gram-positive bacteria; HA: hospital acquired; CA: community acquired; SBP: spontaneous bacterial peritonitis.
